# ZnO nanoparticle-induced oxidative stress triggers apoptosis by activating JNK signaling pathway in cultured primary astrocytes

**DOI:** 10.1186/1556-276X-9-117

**Published:** 2014-03-13

**Authors:** Jieting Wang, Xiaobei Deng, Fang Zhang, Deliang Chen, Wenjun Ding

**Affiliations:** 1 Laboratory of Environment and Health, College of Life Sciences, University of Chinese Academy of Sciences, No. 19A Yuquan Road, Beijing 100049, China

**Keywords:** Zinc oxide nanoparticles, Astrocytes, Oxidative stress, Apoptosis, JNK

## Abstract

It has been documented in *in vitro* studies that zinc oxide nanoparticles (ZnO NPs) are capable of inducing oxidative stress, which plays a crucial role in ZnO NP-mediated apoptosis. However, the underlying molecular mechanism of apoptosis in neurocytes induced by ZnO NP exposure was not fully elucidated. In this study, we investigated the potential mechanisms of apoptosis provoked by ZnO NPs in cultured primary astrocytes by exploring the molecular signaling pathways triggered after ZnO NP exposure. ZnO NP exposure was found to reduce cell viability in MTT assays, increase lactate dehydrogenase (LDH) release, stimulate intracellular reactive oxygen species (ROS) generation, and elicit caspase-3 activation in a dose- and time-dependent manner. Apoptosis occurred after ZnO NP exposure as evidenced by nuclear condensation and poly(ADP-ribose) polymerase-1 (PARP) cleavage. A decrease in mitochondrial membrane potential (MMP) with a concomitant increase in the expression of Bax/Bcl-2 ratio suggested that the mitochondria also mediated the pathway involved in ZnO NP-induced apoptosis. In addition, exposure of the cultured cells to ZnO NPs led to phosphorylation of c-Jun N-terminal kinase (JNK), extracellular signal-related kinase (ERK), and p38 mitogen-activated protein kinase (p38 MAPK). Moreover, JNK inhibitor (SP600125) significantly reduced ZnO NP-induced cleaved PARP and cleaved caspase-3 expression, but not ERK inhibitor (U0126) or p38 MAPK inhibitor (SB203580), indicating that JNK signaling pathway is involved in ZnO NP-induced apoptosis in primary astrocytes.

## Background

In the last decades, zinc oxide nanoparticles (ZnO NPs), as a new type of high-functional nanoparticles, have been widely used in cosmetics, food additives, biosensors, and pharmaceuticals [1-4]. With the increased application of ZnO NPs, the concerns about their potential hazards and safety have also been increased. It has been known that with decreasing particle size, nanoparticles can easily accumulate and migrate deeply in the body. Recently, some studies have demonstrated that inhaled nanoparticles were translocated to the central nervous system through the olfactory neuronal pathway [4-6], resulting in inflammatory changes and brain edema formation [7]. ZnO NPs have been considered as one of the most toxic nanoparticles [8]. Accumulating evidence from *in vitro* studies has revealed that ZnO NPs are toxic to mammalian cells [9-12]. A series of cytotoxic effects exposed to ZnO NPs are observed, such as oxidative damage and cell death [10,12,13].

Oxidative stress has been recognized as an important mechanism underlying the toxic effects of metal oxide NPs, which has been extensively studied *in vitro* and *in vivo*[14-16]. Oxidative stress is definitely caused by an imbalance between production of various reactive oxygen species (ROS) and antioxidant defense [17], and ROS has been identified as signaling molecules in various pathways regulating both cell survival and cell death [18,19]. Excessive levels of ROS, mainly radical form, can cause severe damage to DNA, RNA, and proteins [20]. Recently, a number of previous studies have demonstrated that oxidative stress is involved in ZnO NP cytotoxicity [21-24]. ROS can potently inhibit neurogenesis [25]. It was confirmed that oxidative damage can also affect the glial cells, which are associated with neuronal death or decrease in neuronal proliferation [26]. Oxidative stress and glia-derived ROS are critical for apoptosis-induced selective loss of neurons [27,28]. Exposure to ZnO NPs lead to cytotoxicity in neuro-2A, neural stem cell, and SHSY5Y cells [9,12,29]. However, the triggering mechanisms of ZnO NP-mediated oxidative stress led to apoptotic process in cultured primary neurocytes are poorly understood.

Several signaling transduction pathways, including c-Jun N-terminal kinase (JNK), extracellular signal-related kinase (ERK), and p38 mitogen-activated protein kinase (p38 MAPK), have been implicated in apoptosis [30,31]. JNK is activated by oxidative stress, which modulates cellular functions in the neurons [32]. It has been demonstrated that JNK is involved in the mitochondrial apoptotic pathway in some stress responses [33]. Persistent activation of ERK contributes to oxidative neuronal injury in both neurons and astrocytes [34]. Prolonged activation of ERK triggers glutamate-induced apoptosis of astrocytes [35]. p38 MAPK phosphorylation has also been shown in cultured astrocytes in response to oxidant signaling [36]. However, the molecular mechanisms underlying ZnO NP-related induction of apoptosis have not been fully elucidated.

The purpose of the present study is to delineate the oxidative stress-mediated mechanism by ZnO NPs, which exerts apoptotic effect in cultured primary astrocytes of rats.

## Materials and methods

### Materials

ZnO NPs were obtained from HT Nano Company (Nanjing, China). Dulbecco's modified Eagle's medium/nutrient mixture F12 (DMEM/F12) was purchased from Invitrogen (Carlsbad, CA, USA). Fetal bovine serum (FBS) was obtained from PAA (Pasching, Austria). 3-(4,5-Dimethylthiazol-2-yl)-2,5-diphenyltetrazolium bromide (MTT), *N*-acetylcysteine (NAC), and 2′,7′-dichlorodihydrofluorescein diacetate (H_2_DCFDA) were obtained from Sigma-Aldrich (St. Louis, MO, USA). SP600125 and SB203580 were obtained from Beyotime (Shanghai, China). U0126 was obtained from Promega (Madison, WI, USA). Bicinchoninic acid (BCA) protein assay kit was obtained from Pierce (Rockford, IL, USA). Annexin V-FITC apoptosis kit was purchased from Abcam (Mountain View, CA, USA). Bcl-2, Bax, JNK, ERK1/2, p38 MAPK, phosphor-JNK, phosphor-ERK1/2, phosphor-p38, and caspase-3 antibodies were purchased from Bioworld (St. Louis Park, MN, USA); poly(ADP-ribose) polymerase-1 (PARP) was purchased from Cell Signaling Technology (Boston, MA, USA); β-actin and secondary antibodies (goat anti-mouse or anti-rabbit IgG-conjugated horseradish peroxidase (HRP)) were purchased from Beyotime (Shanghai, China).

### Characterization of ZnO NPs

The shape of ZnO NPs was visualized under a transmission electron microscopy (TEM, CM120, Philips, Amsterdam, the Netherlands) at an accelerating voltage of 100 kV and at least four fields of view.

The average hydrodynamic size of ZnO NPs in water and cell culture medium was determined by dynamic light scattering (DLS; Nano-Zetasizer, 1000 HS, Malvern Instrument Ltd., Worcestershire, UK). Briefly, ZnO NPs was suspended in Milli-Q water and cell culture medium, respectively. The suspensions were sonicated for 30 s at 40 W by ultrasonic processor (VCX130, Sonics & Materials Inc, Newtown, CT, USA).

The surface area of ZnO NPs was determined by multipoint nitrogen adsorption using a Brunauer-Emmett-Teller (BET; ASAP2010, USA).

### Primary cell culture and ZnO NPs treatment

The procedure was modified from preparation of separate astroglial and oligodendroglial cell cultures from rat cerebral tissue as described in the methods of Shahar [37]. In brief, neonatal Sprague-Dawley rats (1-day-old) were obtained from Beijing University Medical Laboratory Animal Center. The brain was removed and transferred to a 60-mm Petri dish and then rinsed with a squirt of modified DMEM/F12 culture medium containing 2 mM of glutamine. The cerebral cortex was gently removed from the individual cortical lobes and then immediately placed into a fresh 60-mm Petri dish. The cortices were dissociated into cell suspension. The cell suspensions were plated in 25-cm^2^ tissue culture flasks at a concentration of 2 × 10^5^ cells/ml. The cells were cultured in DMEM/F12 culture medium in 5% CO_2_ atmosphere at 37°C. Twenty-four hours after the initial plating, the medium was changed to preserve the adhering astrocytes and to remove the neurons and oligodendrocytes. The medium was changed once every 3 days. The astrocytes were maintained in DMEM/F12 containing 10% fetal bovine serum, 100 IU/ml penicillin, and 10 μg/ml streptomycin. The purity of astrocytes was assessed by GFAP-immunostaining according to the Weinstein method [38]. In these conditions, we can assume that over 95% of the cells were astrocytes. All cell exposure experiments were performed at 80% to 90% of cell confluence with viability of ≥90% as determined by trypan blue staining.

The astrocytes (2 × 10^5^ cells/ml) were first cultured in 96-well plates (Costar, Cambridge, MA, USA) for 24 h prior to treatment. Then the culture medium was replaced with serum-free medium, and the cells were exposed to freshly dispersed ZnO NPs preparations at the final concentrations of 4, 8, or 12 μg/ml for 6, 12, or 24 h, respectively. Under the same conditions, astrocytes were pretreated for 1 h with 5 mM of NAC before a 6-h co-exposure with or without ZnO NPs (12 μg/ml). For the inhibitory effect experiments, the cells were pretreated with inhibitor of JNK, ERK, and p38 MAPK pathway (10 μM of SP600125, U0126, and SB203580, respectively) and then treated with ZnO NPs for the indicated duration and concentration.

### Cell viability assay

The effect of ZnO NPs on the viability of astrocytes was measured using the MTT assay according to the method of Deng et al. [39]. After exposure to 4, 8, or 12 μg/ml of ZnO NPs for 6, 12, or 24 h, 10 μl of MTT (5 mg/ml in phosphate buffer solution (PBS)) diluted by 90 μl medium was added to each well and incubated for 1 h at 37°C. The cells were then treated with 100 μl of dimethyl sulfoxide (DMSO). The absorbance was quantified at 570 nm using a microplate spectrophotometer (Thermo MK3, ThermoScientific Instruments, Cambridge, MA, USA). The result was reported as viability with respect to untreated cells.

### SEM and TEM observation

Astrocytes were grown on coverslips to a semiconfluent state and then treated with ZnO NPs at the final concentration of 12 μg/ml. After exposure to 12 μg/ml of ZnO NPs for 6 h, the cells were fixed with 2% glutaraldehyde in 0.1 M cacodylate buffer (pH 7.3), added with 2% sucrose at room temperature for 2 h, then dehydrated using graded ethanol concentrations, critical point-dried in CO_2_, and gold-coated by sputtering. The samples were then examined by scanning electron microscopy (SEM; JSM-6700 F, JEOL Ltd, Tokyo, Japan).

Astrocytes were grown in 50.4-cm^2^ culture dishes and then treated with ZnO NPs at the final concentration of 12 μg/ml for 6 h. The cells were fixed with 2% glutaraldehyde in 0.1 M cacodylate buffered (pH 7.4) at 4°C overnight and then postfixed with 1% OsO_4_ in 0.1 M sodium cacodylate buffer (pH 7.4) at 4°C for 2 h. After dehydration with ascending concentrations of ethanol (50% to 100%), the samples were embedded at 60°C for 2 days. Ultrathin sections (80 nm) were stained with uranyl acetate and lead citrate. The sections were examined by transmission electron microscopy (CM120, Philips, Netherlands) at 80 kV.

### LDH assay

The level of lactate dehydrogenase (LDH) released from astrocytes was measured to evaluate the cytotoxicity of ZnO NPs. Briefly, astrocytes were treated with 4, 8, or 12 μg/ml of ZnO NPs for 6, 12, or 24 h, respectively. The cell-free supernatant was separated by centrifuge (2,000 rpm, 5 min). The culture supernatants were transferred to clean flat-bottom plate for enzymatic analysis. The activity of LDH in the supernatants was determined using LDH detection kit (Nanjing Jiancheng Bioengineering Institute, Nanjing, China) according to the manufacturer's instructions. All samples were assayed in duplicates for LDH content by a microplate spectrophotometer (Thermo MK3, MA, USA).

### ROS assay

The intracellular levels of ROS were determined by measuring the oxidative conversion of DCFH-DA to fluorescent compound dichlorofluorescin (DCFH) [40]. Briefly, astrocytes were placed in 24-well for 12 h, then treated with 4, 8, or 12 μg/ml of ZnO NPs for 6, 12, or 24 h, incubated with DCF diacetate in culture medium for 15 min, and washed with cold phosphate buffer solution three times. The measurement of green fluorescence (oxidized DCFH) using a microplate fluorometer (LB 941, Berthold Technologies, Bad Wildbad, Germany) with fluorescence intensity (excitation, 488 nm; emission, 530 nm). The total protein concentration was determined using BCA protein assay kits (Pierce, IL, USA). The cell-free wells containing only ZnO NPs and DCFH were used to assess nonspecific particle-induced fluorescence. Fluorescence was reported with respect to unexposed control cells.

### Assessment of mitochondrial membrane potential

Mitochondrial membrane potential (MMP) was determined with JC-1, a lipophilic membrane-permeable cationic probe, which is widely used for detecting MMP [41]. After 6, 12, or 24 h exposure to 4, 8, or 12 μg/ml of ZnO NPs, the cells were washed twice in PBS and were incubated for 30 min at 37°C with JC-1, a lipophilic and cationic dye that accumulates in the mitochondria in a potential-dependent manner. The intensity of fluorescence was measured with a fluorescence multi-well plate reader LB 941 (Berthold, Bad Wildbad, Germany) with fluorescence intensity (green: excitation 488 nm, emission 530 nm; red: excitation 535 nm, emission 605 nm). The results were presented as the ratio of intensity of red and green fluorescence.

### Nuclear staining with DAPI

Chromatin condensation was determined by 4, 6-diamido-2-phenylindole dihydrochloride (DAPI) staining. Astrocytes were cultured in DMEM containing 10% fetal bovine serum on poly-l-lysine-coated dishes or slides in 5% CO_2_, at 37°C then treated with 12 μg/ml concentration of ZnO NPs for 6 h. The cells were collected and sequentially washed three times in PBS and then fixed for 15 min in 4% paraformaldehyde in PBS for 30 min at room temperature. After staining with a DAPI solution (10 μg/ml) for 10 min in the dark at room temperature, the stained cells were washed twice with PBS to remove the excess DAPI and the changes of nucleus were examined with excitation of 330 to 380 nm and emission of 420 nm using a fluorescence microscope (Olympus, Tokyo, Japan).

### Flow cytometric analysis

Apoptosis was measured using the Annexin V-FITC apoptosis detection kit (Abcam, Mountain View, CA, USA) according to the manufacturer's instructions. After treatment with 4, 8, or 12 μg/ml of ZnO NPs for 6 h, the cells were harvested with trypsin, washed twice with PBS (pH 7.4), and then incubated with 200 μl of binding buffer containing Annexin V-FITC (40 μl/ml) and propidium iodide (PI; 1 μg/ml) for 15 min at room temperature in the dark. The population of Annexin V-positive cells was analyzed by flow cytometry (Epics XL, Beckman Coulter Inc, Pasadena, CA, USA). The early apoptotic cells were located in the lower right quadrant (Annexin V-FITC-positive/PI-negative cells), and the late apoptotic cells were located in the upper right quadrant (Annexin V-FITC-positive/PI-positive cells). The percentages of apoptotic cells (Annexin V-positive cells) were plotted.

### Western blot analysis

The expression of Bcl-2, Bax, JNK, ERK1/2, PARP, p38 MAPK, phosphor-JNK, phosphor-ERK1/2, phosphor-p38 MAPK, cleaved caspase-3, and β-actin in whole cell lysates were analyzed by sodium dodecyl sulfate polyacrylaminde gel electrophoresis (SDS-PAGE). The gels were transferred to polyvinylidene difluoride (PVDF) membrane by semi-dry electrophoretic transfer at 20 V for 60 min using the semi-dry transfer system. The PVDF membranes were blocked with 5% nonfat milk at room temperature for 1 h, incubated with the primary antibody (dilution 1:1,000) in Tris/buffered saline/Tween20 (TBST) containing 5% bovine serum albumin overnight in 4°C, and then incubated with the secondary antibody (dilution 1:1,000) at room temperature for 1 h. Immunoreactive bands were detected by an enhanced chemiluminescence detection kit (Millipore Corporation, Billerica, MA, USA) according to the manufacturer's instructions. β-actin was used as loading controls for the total protein content and showed no differences between groups.

### Statistical analysis

Results were presented as mean ± standard deviation (SD) of three representative experiments. Data were analyzed using one-way analysis of variance (ANOVA) followed by *post hoc* comparisons using the Dunnet's multiple comparison test or two-way ANOVA statistical analysis followed by a *post hoc* test. A probability of value of *p* < 0.05 was considered as statistically significant.

## Results

### Characterization of ZnO NPs

**Figure 1:**
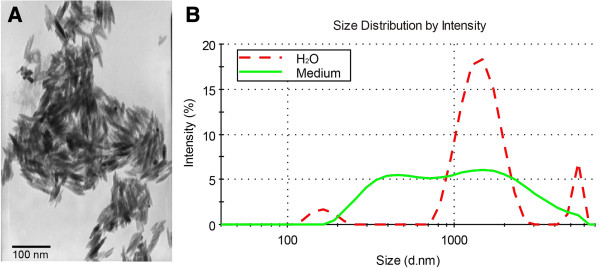
**Characterization of ZnO nanoparticles. (A)** Transmission electron microscope (TEM) image of ZnO NPs. Scale bars, 100 nm. Magnification × 100,000. **(B)** Size distributions of ZnO NPs in H_2_O or DMEM/F12 were analyzed by dynamic light scattering, respectively.

Figure 1A shows the TEM images of ZnO NPs. The picture exhibits that the majority of ZnO NPs were rod-shaped with smooth surfaces. The average TEM size of ZnO NPs was about 45 nm. The surface area of ZnO NPs determined by BET was 35 m^2^/g. The average hydrodynamic size of ZnO NPs in water and cell culture medium determined by DLS was 2,181 and 747 nm, respectively. The physicochemical characteristics of ZnO NPs are listed in Table 1.

**Table 1:** Physicochemical characteristics of ZnO NPs

**Parameters**	**Values (mean ± SD)**
Average TEM size (nm)	45 ± 27
Hydrodynamic size in distilled water (nm)	2181 ± 21
Hydrodynamic size in culture medium (nm)	747 ± 25
Surface area (m^2^/g)	35.34 ± 2.82

Description: zinc oxide, nanopowder.

### ZnO NP-induced cytotoxicity

**Figure 2:**
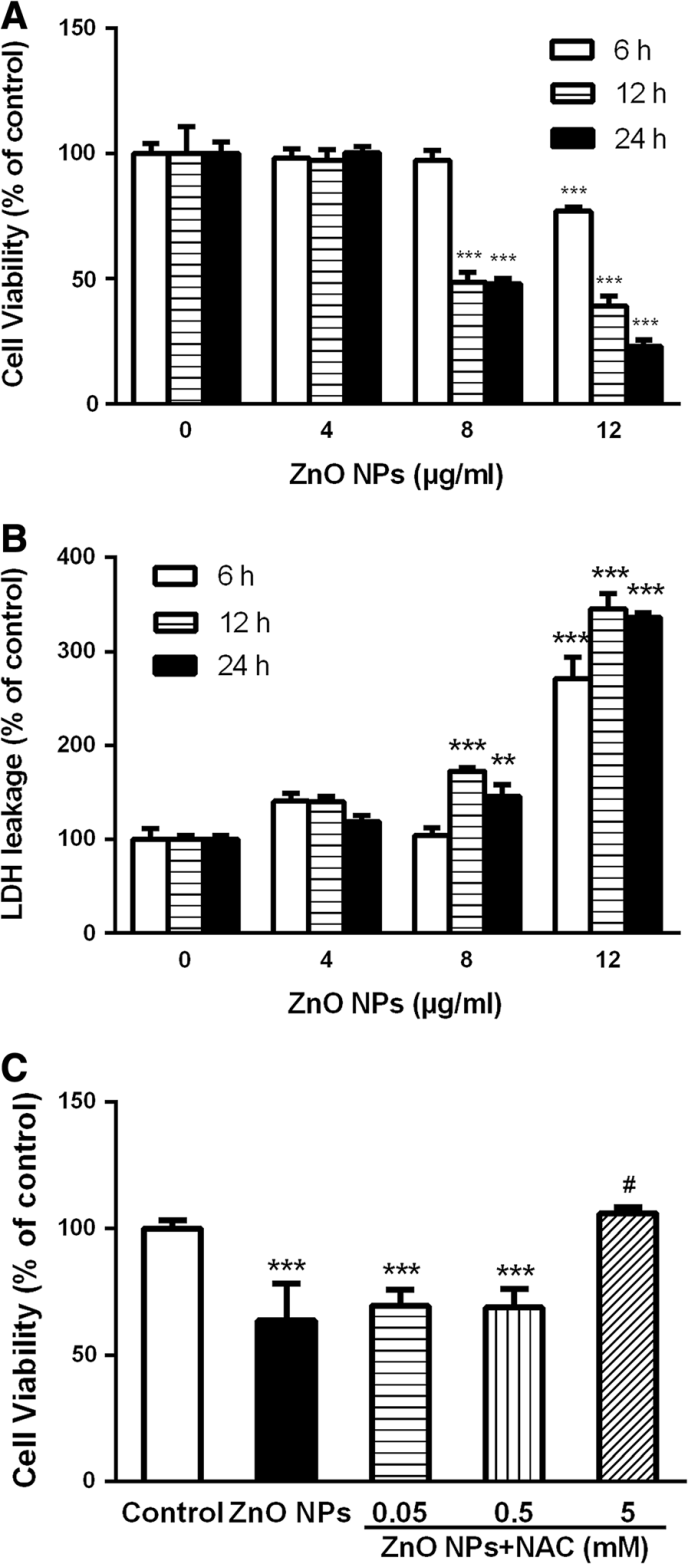
**Effect of ZnO NPs on cell viability in cultured primary astrocytes. (A)** MTT assay. **(B)** LDH release. Astrocytes were treated with 4, 8, or 12 μg/ml of ZnO NPs for 6, 12, or 24 h, respectively. **(C)** Inhibitory effect of *N*-acetylcysteine (NAC) on cell viability reduction. Astrocytes were pretreated for 1 h with 0.05, 0.5, and 5 mM of NAC before 6 h co-exposure with ZnO NPs (12 μg/ml). Data are presented as mean ± SD of the three representative experiments. **p < 0.05, ***p < 0.001 vs. control. #*p* < 0.05 vs. ZnO NPs (12 μg/ml).

The cytotoxicity of ZnO NPs in astrocytes was evaluated with the MTT and LDH assays. The cell viability examined by MTT assay showed no statistically significant impacts of ZnO NPs after 6, 12, or 24 h exposure of cells to 4 μg/ml of ZnO NPs compared with the unexposed control cells. However, a significant decrease of cell viability was observed after 12 or 24 h exposure of cells to 8 or 12 μg/ml of ZnO NPs (Figure 2A).

Compared with the unexposed control cells, the level of LDH released from the cells was significantly increased after 12 or 24 h of exposure to 8 or 12 μg/ml of ZnO NPs in dose- and time-dependent manner (Figure 2B). The results indicate that the amount of LDH released from the cells induced by exposure to ZnO NPs is related to the cell viability. As shown in Figure 2C, we also clearly observed a significant inhibitory effect of NAC (5 mM) on cell viability reduction (*p* < 0.05).

### ZnO NPs caused cellular morphological modifications

**Figure 3:**
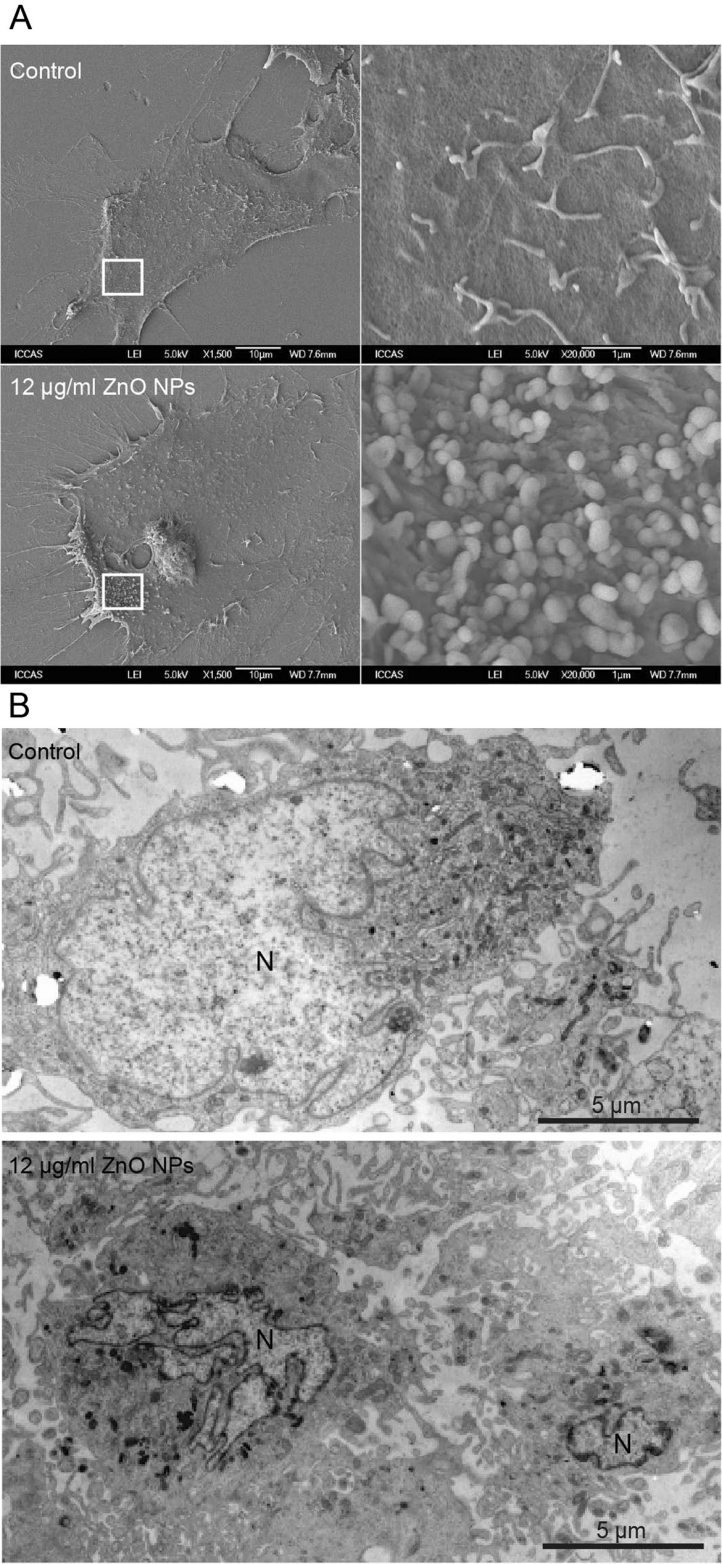
**SEM and TEM images of astrocytes exposed to ZnO NPs for 6 h. (A)** SEM images. Scale bars, 10 and 1 μm. Magnification × 1,500 (left) and × 20,000 (right). **(B)** TEM image. N indicates nucleus. Scale bar, 5 μm. Magnification × 6,000.

To further determine whether ZnO NPs could result in damage in astrocytes, we decided to observe the morphological changes at the ultrastructural level. As shown in Figure 3A, the unexposed control cells remained smooth and flat and also exhibited a cell surface evenly covered by microvillar structures that are well extended with small lamellapodia, indicating the fluidity and motility of normal cells. However, ZnO NP-treated astrocytes exhibited the conformation of numerous lamellapodia and filopodia that were shown projecting from the cell membranes over the substratum, which were probably caused by the aggregation of ZnO NPs in random clusters at the cell surface (Figure 3A). As shown in Figure 3B, ZnO NP-exposed cells displayed typical apoptotic features, including nuclear shrinkage, chromatin condensation, and extensive cytoplasmic vacuolization after 6 h of exposure to 12 μg/ml of ZnO NPs as compared to the unexposed control cells. In addition, we noted that the engulfed ZnO NPs were visualized in the damaged astrocytes.

### ZnO NP-induced intracellular ROS generation

**Figure 4:**
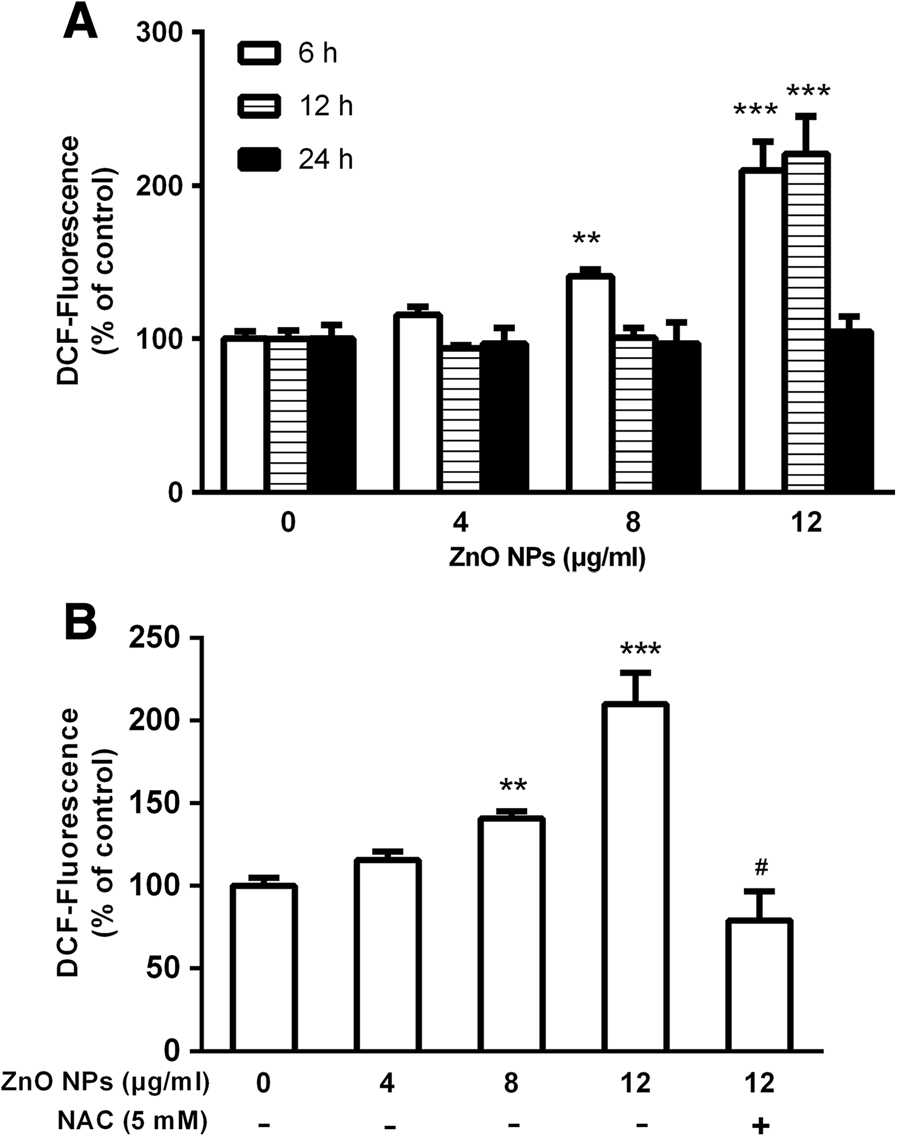
**Effect of ZnO NPs on intracellular ROS generation in cultured primary astrocytes. (A)** ROS generation in astrocytes. Cells were treated with 4, 8, or 12 μg/ml of ZnO NPs for 6, 12, or 24 h, respectively. **(B)** Inhibitory effect of NAC on ROS generation. Astrocytes were pretreated for 1 h with 5 mM of NAC before 6 h of co-exposure with ZnO NPs (12 μg/ml). Data are presented as the mean ± SD of three representative experiments. **p < 0.01, ***p < 0.001 vs. control. #*p* < 0.05 vs. ZnO NPs (12 μg/ml).

The level of ZnO NP-induced intracellular ROS generation was measured by DCF fluorescence intensity in astrocytes. As shown in Figure 4A, a dose-dependent increase of intracellular ROS generation was observed after 6 h of exposure to 8 and 12 μg/ml of ZnO NPs. Moreover, we also found that after 12 h of exposure to 12 μg/ml of ZnO NPs, the level of ROS was significantly higher than that of the unexposed control cells (*p* < 0.001). However, treatment with NAC significantly reduced ZnO NP-induced ROS generation (Figure 4B).

### ZnO NP-induced mitochondrial dysfunction

**Figure 5:**
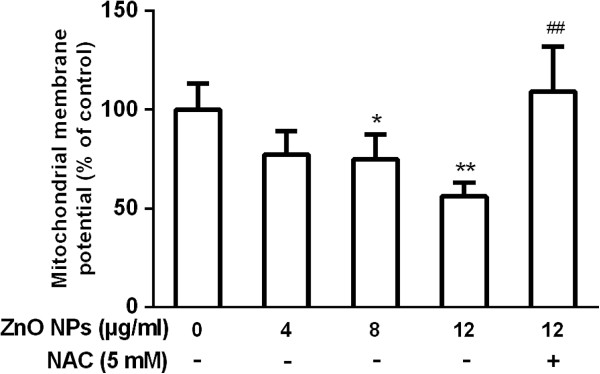
**Effect of ZnO NPs on mitochondrial transmembrane potential in cultured primary astrocytes.** The cells were treated with 4, 8, or 12 μg/ml of ZnO NPs or 12 μg/ml of ZnO NPs plus 5 mM NAC for 6 h, respectively. Astrocytes were pretreated for 1 h with 5 mM of NAC before 6 h of co-exposure with ZnO NPs (12 μg/ml). Data are presented as the mean ± SD of three representative experiments. *p < 0.05, **p < 0.01, ***p < 0.001 vs. control. #*p* < 0.05 vs. ZnO NPs (12 μg/ml).

Healthy cells with functional mitochondria color stain with red JC-aggregates while cells with impaired mitochondria stain with green JC-1 monomers. As shown in Figure 5, ZnO NP-exposed cells exhibited a marked impairment of mitochondria in a dose-dependent manner evident by a shift in JC-1 florescence from red to green. However, the MMP level was increased up to control level for ZnO NPs in the presence of NAC (Figure 5).

### ZnO NP-modulated intrinsic apoptosis

**Figure 6:**
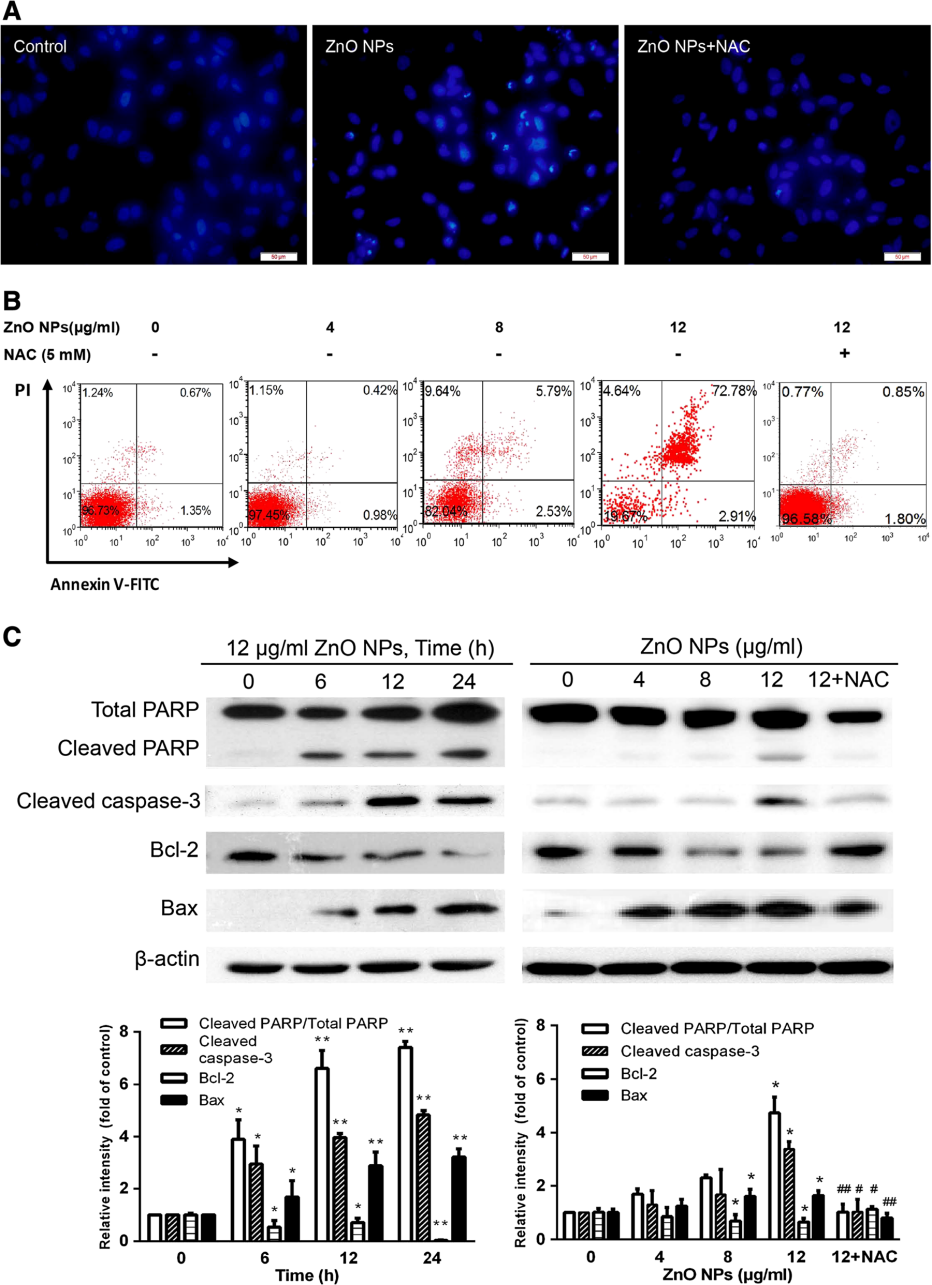
**Effect of ZnO NPs on apoptosis in cultured primary astrocytes. (A)** Fluorescent images of chromosome condensation by DAPI staining. Control cells (left), cells exposed to 12 μg/ml of ZnO NPs for 6 h (middle). Cells were pretreated for 1 h with 5 mM of NAC before 6 h co-exposure with 12 μg/ml ZnO NPs (right). Scale bar, 50 μm. Magnification, ×400. **(B)** FACS results of the Annexin V-FITC and PI assays. Cells were treated with 4, 8, or 12 μg/ml of ZnO NPs or 12 μg/ml of ZnO NPs plus 5 mM of NAC for 6 h, respectively. The astrocytes were pretreated for 1 h with 5 mM of NAC before 6 h of co-exposure with ZnO NPs (12 μg/ml). The dot plots and the relative mean values have been obtained from a single representative experiment of three representative experiments that gave very similar results. **(C)** Effect of ZnO NPs on the expression of apoptosis-related proteins in cultured primary astrocytes. The cells were treated with 4, 8, or 12 μg/ml of ZnO NPs or 12 μg/ml of ZnO NPs plus 5 mM of NAC for 6 h or 12 μg/ml of ZnO NPs for 6, 12, or 24 h, respectively. The cells were collected to measure their protein expression levels (cleaved PARP, cleaved caspase-3, Bcl-2, Bax, and β-actin) as described in the ‘Materials and methods’ section. Data are presented as the mean ± SD of the three representative experiments. *p < 0.05, **p < 0.01, ***p < 0.001 vs. control. #*p* < 0.05, ##*p* < 0.01 vs. ZnO NPs (12 μg/ml).

As shown in earlier, ZnO NPs markedly decreased MMP, thus we further examined the levels of the key apoptotic and signaling proteins involved in the mitochondrial pathway of apoptosis in ZnO NP-exposed cells. As shown in Figure 6A, ZnO NP-exposed astrocytes displayed chromatin condensation as compared with unexposed control cells. Moreover, as the exposure concentration of ZnO NPs was increased from 4 to 12 μg/ml, the total number of apoptotic cells increased from 5.79% to 72.78% (Figure 6B). Furthermore, the protein levels of Bax, cleaved PARP, and cleaved caspase-3 were significantly up-regulated while the expression of Bcl-2 was significantly downregulated in ZnO NP-exposed cells (Figure 6C). Correspondingly, Western blotting data showed that pretreatment with NAC not only significantly suppressed downregulation of Bcl-2 and up-regulation of Bax but also attenuated cleaved PARP and cleaved caspase-3 expression induced by ZnO NPs (Figure 6C).

### Involvement of JNK/ERK/p38 MAPK signaling pathways in the ZnO NP-induced apoptosis

To determine whether JNK/ERK/p38 MAPK signaling pathways are involved in ZnO NP-induced apoptosis, we firstly detected the phosphorylation of JNK, ERK, and p38 MAPK after exposure of astrocytes to 12 μg/ml of ZnO NPs for 0, 6, 12, or 24 h. As shown in Figure 6A, ZnO NPs rapidly and markedly increased JNK phosphorylation at 6 and 12 h after exposure. The phosphorylation of ERK was significantly increased from 6 to 24 h after exposure. Moreover, there was a marked increase in p38 MAPK phosphorylation from 6 to 24 h. In addition, when the cells were treated with 4, 8, or 12 μg/ml of ZnO NPs for 6 h, phosphorylation of induction of JNK, ERK, and p38 MAPK seemed to be induced maximally after exposure to ZnO NPs (12 μg/ml). These findings indicated that ZnO NP exposure induces phosphorylation of JNK, ERK, and p38 MAPK, respectively.

To confirm that the underlying mechanism of JNK/ERK/p38 MAPK signaling pathways is involved in ZnO NP-induced apoptosis, we next utilized JNK/ERK/p38 MAPK inhibitors after exposure of astrocytes to 12 μg/ml of ZnO NPs for 6 h. As shown in Figure 6B, 10 μM of JNK inhibitor (SP600125) significantly downregulated the expression of cleaved PARP and cleaved caspase-3 after exposure to ZnO NPs (*p* < 0.001). However, pretreatment with either ERK 1/2 inhibitor (10 μM PD98059) or p38 MAPK inhibitor (10 μM SB203580) did not affect the expression of cleaved PARP and cleaved caspase-3. In addition, we also found that LY294002 significantly downregulated ZnO NP-induced cleaved PARP and cleaved caspase-3 expression rather than SB203580 and PD98059 (Figure 6B). These results suggested that ZnO NP-induced apoptosis is mediated by the JNK signaling pathway.

**Figure 7:**
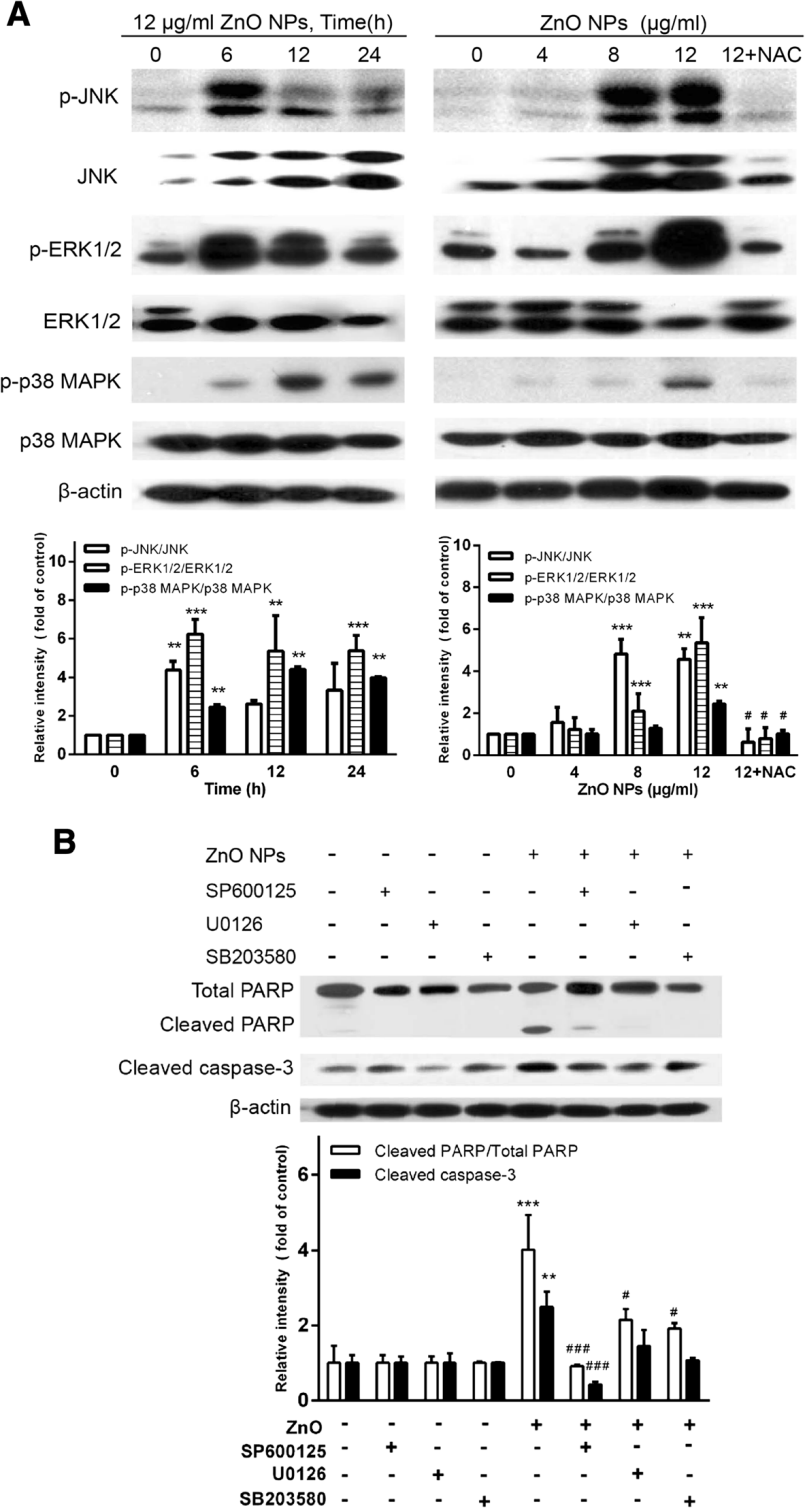
**Effect of ZnO NPs on ROS-dependent JNK/ERK/p38 MAPK activation in cultured primary astrocytes. (A)** Cells were treated with 4, 8, or 12 μg/ml of ZnO NPs or 12 μg/ml of ZnO NPs plus 5 mM NAC for 6 h or 12 μg/ml of ZnO NPs for 6, 12, or 24 h, respectively. Cell lysates were blotted for phospho-JNK, JNK, phospho-ERK1/2, ERK1/2, phospho-p38 MAPK, and p38 MAPK, respectively. The density of the phosphorylated-JNK/ERK1/2/p38 MAPK was relative to the level of JNK/ERK1/2/p38 MAPK, respectively. **(B)** Cells were pretreated with the specific JNK inhibitor (10 μM SP600125), ERK inhibitor (10 μM U0126) or p38 MAPK inhibitor (10 μM SB203580) for 1 h and then treated with 12 μg/ml ZnO NPs for 6 h, respectively. The cell lysates were blotted for total PARP, cleaved PARP, and cleaved caspase-3. The density of cleaved caspase-3 was relative to the level of β-actin. Density of cleaved PARP was relative to the level of total PARP. Data are presented as the mean ± SD of three representative experiments. **p < 0.01 and ***p < 0.001 vs. control. #*p* < 0.05 and ###*p* < 0.001 vs. ZnO NPs (12 μg/ml).

To assess whether ZnO NP-induced ROS generation was involved in the JNK signaling pathway in astrocytes, we further detected the phosphorylation of JNK after exposure to ZnO NPs in the presence of NAC (5 mM) for the indicated duration. As shown in Figure 7A, phosphorylation of JNK was also markedly decreased after exposure to ZnO NPs in the presence of NAC. Moreover, cleaved PARP expression was significantly downregulated after pretreatment with NAC. These findings indicated that the JNK signaling pathway is involved in ROS-induced apoptosis in response to ZnO NPs.

## Discussion

Nanoparticles are associated with neuroinflammation, neurodegeneration, and increased risk for neurodegenerative diseases [42-44]. These associations are related to the capability of nanoparticles to enter into the olfactory bulb and to deposit in the central nervous system [6]; however, the exact mechanism involved in the cytotoxicity is not fully described. Several studies have identified the oxidative stress as common pathway for NP-induced damage [14,23,45-47]. Recently, Guan et al. demonstrated that ZnO NPs are capable of inducing oxidative stress responses in human hepatocyte and embryonic kidney cells [21]. In the present study, we showed that exposure to ZnO NPs causes morphological alterations and cytotoxicity. Moreover, ZnO NPs trigger an increase of intracellular ROS in a dose-dependent manner, followed by chromosome condensation, MMP depolarization, JNK/ERK/p38 MAPK phosphorylation, and PARP cleavage in cultured primary astrocytes. Furthermore, pretreatment with JNK inhibitor (SP600125), but not ERK inhibitor (U0126) or p38 MAPK inhibitor (SB203580), significantly reduced ZnO NP-induced cleaved PARP and cleaved caspase-3 expression, indicating that JNK signaling pathway is involved in ZnO NP-induced apoptosis.

Accumulating evidences have indicated that nanoparticles can induce neuron and glia cell apoptosis by targeting the mitochondrial apoptosis pathway, which includes activation loop phosphorylation, cytochrome c release from the mitochondria, decrease in Bcl-2 protein expression, activation of PARP and caspase cascades, and DNA fragmentation [10,12,44,48,49]. Here, our data also showed that ZnO NP-induced oxidative stress followed by significant reduction of the MMP and modifications of Bax and Bcl-2 protein expression with an increase of Bax levels and a corresponding decrease in Bcl-2 levels (Figure 5A,B), leading to caspase-3 activation and PARP cleavage. This observation is in agreement with an earlier report demonstrating that ZnO NP exposure induced apoptotic cell death via p53, survivin, Bax/Bcl-2, and caspase pathways mediated by oxidative stress [50].

Oxidative stress is the result of an imbalance in the pro-oxidant/antioxidant homeostasis. Various ROS, such as superoxide, hydrogen peroxide, hydroxyl, and other oxygen radicals, are involved in oxidative stress. ROS function as signaling molecules in various pathways regulating both cell survival and cell death [19]. ROS is also reportedly associated with neurodegenerative disorders, such as Alzheimer's disease, Parkinson's disease, and Huntington's disease [44]. Generation of ROS is considered to be up-stream event for the initiation of NP-induced apoptotic signaling *in vitro*[51,52]. It has been reported that the oxidative stress is a common pathway for ZnO NP-induced oxidative damage [1,50,53]. Previous studies have identified mitochondria as the main sources of ROS [54,55]. Others have shown that ROS cause a decrease of the MMP and initiate mitochondria-mediated apoptosis [56,57]. The cytotoxicity and oxidative stress exerted by ZnO NPs and derived also from their oxidative potential are probably associated with the nanolevel characteristic of ZnO NPs, disorder of electron transport chain in mitochondria, and production of reduced nicotinamide phosphate dinucleotide (NADPH) [45,58,59]. Here, our data showed that ZnO NP-induced ROS triggered apoptosis and decreased the MMP because ROS scavenger NAC markedly inhibited the processes (Figures 4B and 5). Therefore, it is conceivable that ZnO NP-induced ROS triggers a decrease of mitochondria membrane potential that leads to apoptosis in astrocytes.

Sharma et al. described that the MAPK pathway regulates apoptosis in response to ZnO NPs [45]. It is also well known that JNK is an important regulator of cell apoptosis and survival in response to oxidative stress [30,60]. In the present study, we evaluated if the JNK, ERK1/2, and p38 MAPK pathways were involved in regulating ZnO NP-induced apoptosis. We found that ZnO NPs induce a time- and dose-dependent increase in the phosphorylation of JNK, ERK, and p38 MAPK, which indicates that ZnO NPs enhance the activities of these three stress-activated protein kinases. However, treatment using the selective MAPK inhibitors SP600125, U0126, and SB203580 significantly resulted in the suppression of the PARP activation and attenuated caspase-3 activity after exposed to ZnO NPs, whereas SB203580 had no significant effects. Thus, we propose that JNK pathway is involved in the apoptotic process in primary astrocytes induced by ZnO NPs.

On the other hand, we also observed that NAC partially suppressed the activation of JNK after ZnO NP exposure, even when the oxidative stress is decreased. The complementary evidence indicated that ROS generation is associated with the activation of the JNK pathway, which contributes to PARP and caspase-3 activation after exposure to ZnO NPs. Wu et al. described that MAPK pathway regulates apoptosis induced by oxidative stress in response to TiO_2_ nanoparticles in neuron cells [49]. Recently, it has been demonstrated that TiO_2_ nanoparticles activate the MAPK signaling pathway and induced caspase-3-mediated PARP cleavage [61].

## Conclusions

In summary, the present study demonstrated that ZnO NP-induced oxidative stress activates JNK signaling pathway, compromising the integrity of cellular membranes and leading to apoptosis of astrocytes. However, more investigations are needed to further explore the role of apoptosis in ZnO NP-induced cytotoxicity and pathogenesis of neurodegenerative diseases associated with ZnO NP exposure.

## Abbreviations

DAPI: 4, 6-diamido-2-phenylindole dihydrochloride; DCFH-DA: 2′, 7′-dichlorofluorescein diacetate; MAPK: mitogen-activated protein kinases; NAC: *N*-acetylcysteine; PI: propidium iodide; ROS: reactive oxygen species; SEM: scanning electron microscopy; TEM: transmission electron microscopy.

## Competing interests

The authors declare that they have no competing interests.

## Authors’ contributions

JW substantially carried out the whole of experiments, performed the statistical analysis, and drafted the manuscript. XD participated in the manuscript editing. FZ and DC partly participated in its coordination. WD conceived the study and participated in its design and revised the manuscript. All authors read and approved the final manuscript.
